# Secondary Metabolites from the Red Macroalga *Sphaerococcus coronopifolius* as Hit Candidates of *Helicobacter pylori* Purine Nucleoside Phosphorylase

**DOI:** 10.1021/acsomega.6c05168

**Published:** 2026-10-08

**Authors:** Ana Micaela Camini, Maria Eduarda Delawi, Bruno Rampanelli Dahmer, Manuella Silva da Costa, Celso Miguel da Maia Alves, Joana Rita Martins da Silva, Rui Filipe Pinto Pedrosa, Jeferson Camargo de Lima, Luís Fernando Saraiva Macedo Timmers

**Affiliations:** † Universidade do Vale do Taquari — Univates, Lajeado 95914-014, Rio Grande do Sul, Brazil; ‡ Programa de Pós-Graduação em Biotecnologia (PPGBiotec), Universidade do Vale do Taquari – Univates, Lajeado 95914-014, Rio Grande do Sul, Brazil; § MARE—Marine and Environmental Sciences Centre, ARNET—Aquatic Research Network, Politécnico de Leiria, 2520-630 Peniche, Portugal; ∥ Programa de Pós-Graduação em Ciências Médicas (PPGCM), Universidade do Vale do Taquari – Univates, Lajeado 95914-014, Rio Grande do Sul, Brazil

## Abstract

*Helicobacter pylori* is a major human pathogen associated with gastric cancer and a growing global public health concern due to increasing antimicrobial resistance. As *H. pylori* lacks *de novo* purine biosynthesis and relies entirely on purine salvage for nucleotide production, purine nucleoside phosphorylase represents a rational antibacterial target. Natural products remain a valuable and largely underexplored source of structurally diverse scaffolds for the discovery of novel enzyme inhibitors, with marine organisms representing a rich yet underexploited source of such bioactive metabolites; however, the pharmacological exploration of this enzyme from natural sources remains limited. In this context, six terpenoids, including five brominated diterpenes, isolated from the red macroalga *Sphaerococcus coronopifolius* were screened against recombinant *H. pylori* PNP using a target-based biochemical approach. Two of the six compounds tested, bromosphaerol and sphaerodactylomelol, showed reproducible inhibitory activity at 30 μM, reducing *H. pylori* PNP activity by 46.04 ± 4.70% and 39.39 ± 8.24%, respectively. Molecular docking predicted binding of both compounds within the active-site purine-binding region, with hydrophobic contacts involving Phe159 and Leu206. Hydrogen bonds were predicted with the backbone carbonyl of Ile178 for bromosphaerol and of Phe159 for sphaerodactylomelol. Molecular dynamics simulations (200 ns, three independent replicates per system) showed that both ligands remained stably accommodated within the predicted active-site binding pocket across all six monomers of the hexameric assembly, while root mean square deviation (RMSD) and root mean square fluctuation (RMSF) analyses indicated that the enzyme retained its global structural integrity throughout the simulations. Bromosphaerol exhibited a lower RMSD than the conformationally flexible sphaerodactylomelol, reflecting differences in ligand dynamics within the binding pocket. Taken together, these findings provide initial biochemical evidence that *S. coronopifolius* represents a source of secondary metabolites capable of interacting with *H. pylori* PNP, and nominate bromosphaerol and sphaerodactylomelol as preliminary hits for future concentration–response characterization.

## Graphical abstract

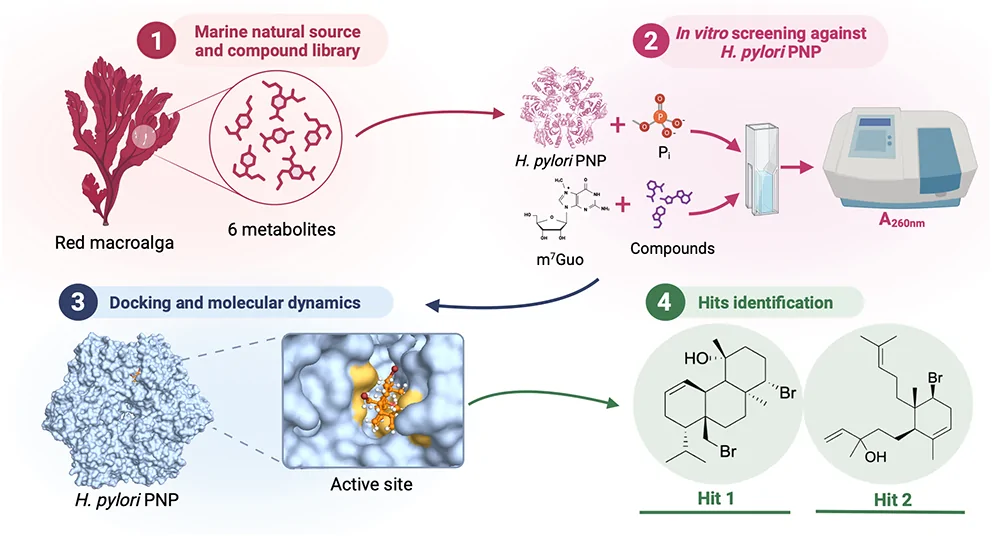

## Introduction

*Helicobacter pylori* is the bacterial pathogen most strongly associated with cancer development, recognized by the International Agency for Research on Cancer (IARC) as a Group I carcinogen and the leading etiological agent of gastric cancer, which is one of the five most common cancers and a leading cause of cancer-related mortality worldwide.^1–3^ Beyond this carcinogen potential, *H. pylori* is also one of the most prevalent chronic bacterial infections in humans, remaining a major public health concern worldwide.^4^ Epidemiological data report prevalence exceeding 50% in parts of Asia, approaching 80% in some African settings, and reaching 60% in Latin America and the Caribbean, disproportionately affecting low- and middle-income countries with inadequate sanitation infrastructure.^4,5^

Given that approximately three-quarters of gastric cancer cases are preventable through eradication of infection,^6^ *H. pylori* eradication has become a central component of clinical management and cancer prevention strategies.^7,8^ Current standard-of-care regimens include clarithromycin-based triple therapy and bismuth-containing quadruple therapy, with regimen selection depending on local antimicrobial resistance patterns and previous treatment history.^5^ However, the effectiveness of these antibiotic-based regimens has been progressively undermined by the global emergence and spread of antimicrobial resistance.^9,10^

Regional surveillance data indicate that primary clarithromycin resistance exceeds 20% in several European regions and reaches as high as 34% in the Western Pacific, while metronidazole resistance surpasses 50% across multiple regions, including Africa, the Eastern Mediterranean, and the Western Pacific.^9^ As a result, eradication rates frequently fall below the 80% threshold considered clinically acceptable, and salvage regimens show markedly reduced efficacy when the same antibiotic class is repeated after first-line failure.^5,9^ This growing challenge has intensified the search for alternative therapeutic approaches, including the identification of novel bioactive compounds capable of targeting essential bacterial processes with mechanisms of action distinct from those of the currently used antibiotics.^5,11^

*H. pylori* lacks the *de novo* purine biosynthesis pathway and relies entirely on the purine-salvage pathway to sustain nucleotide production.^12^ Within this pathway, purine nucleoside phosphorylase (PNP) plays a central role by catalyzing the reversible phosphorolysis of purine nucleosides, thereby supporting DNA and RNA synthesis.^13^ The genetic essentiality of PNP for *H. pylori* viability is supported by deletion of the *deoD* gene, which compromises bacterial growth under purine-limiting conditions,^13^ a finding further corroborated pharmacologically by studies showing that small-molecule inhibition of PNP similarly impairs bacterial growth in culture.^14,15^

Structurally, *H. pylori* PNP belongs to the homohexameric class of purine nucleoside phosphorylases, which are widespread among bacteria and generally exhibit broader substrate specificity than the homotrimeric PNPs found in mammals. Hexameric and trimeric PNPs differ substantially in their oligomeric organization, substrate specificity, active-site architecture, and the residues involved in substrate recognition.^16,17^ These structural and functional differences have been successfully exploited in other bacterial PNPs, where substrates and inhibitors designed for the hexameric enzyme show minimal cross-reactivity with the trimeric human homologue, as demonstrated in gene-directed enzyme prodrug therapy strategies using *E. coli* PNP.^17,18^ Although comparable selective inhibitors have not yet been developed for *H. pylori* PNP, the structural differences between bacterial and human PNPs support the feasibility of developing selective inhibitors.

Despite this favorable structural rationale, the chemical space of *H. pylori* PNP inhibitors derived from natural sources remains largely unexplored; characterized inhibitors to date have been restricted to synthetic purine analogues, particularly 2,6-disubstituted purines and related nucleoside mimetics.^14,15^ The identification of structurally distinct, non-nucleosidic scaffolds from natural sources would, therefore, represent a complementary approach to expanding the inhibitor landscape for this target.

Alongside advances in target-based drug discovery, natural products have remained a valuable source of structurally diverse bioactive molecules.^19^ In particular, the marine environment has gained recognition as a chemically diverse reservoir of secondary metabolites shaped by intense ecological and evolutionary pressures, often displaying unique structural features and a strong capacity to interact with a broad range of biological targets. Although most approved marine-derived drugs have been developed for oncological applications, their chemical diversity also offers significant opportunities for the discovery of new antimicrobial agents, a comparatively underexplored dimension of marine bioprospecting.^20,21^ Within this context, the red macroalga *Sphaerococcus coronopifolius* has attracted considerable interest as a recognized source of brominated diterpenes, several of which have shown bioactivity in different biological systems.^22–24^ Nevertheless, the compounds of *S. coronopifolius* have not been evaluated against defined enzymatic targets, with existing studies limited to phenotypic assays against bacterial pathogens other than *H. pylori*, computational predictions, or cytotoxicity assessments.^25–27^ No previous study has investigated the activity of *S. coronopifolius*-derived metabolites against *H. pylori* or against *H. pylori* PNP specifically.

To address this gap, six terpenoids, including five brominated diterpenes, isolated from *S. coronopifolius*, were evaluated for their potential to modulate *H. pylori* PNP activity using a target-based biochemical approach. By focusing on chemically defined compounds rather than crude extracts, this work provides preliminary biochemical evidence of the interaction between *S. coronopifolius*-derived metabolites and *H. pylori* PNP. These findings expand the knowledge of the biological activities associated with marine-macroalgae-derived compounds and support further investigations into their effects on bacterial metabolic targets.

## Results and Discussion

A validated biochemical assay based on recombinant *H. pylori* PNP enzyme was employed to evaluate the inhibitory potential of chemically defined terpenoids isolated from the red macroalga *Sphaerococcus coronopifolius* (Figure 1). For this purpose, recombinant *H. pylori* PNP was produced using the *E. coli* BL21­(DE3)­pLysS strain as the expression system. Its construct included a C-terminal polyhistidine tag to enable purification by Ni–NTA affinity chromatography, yielding approximately 0.8 mg of purified protein per liter of bacterial culture. Protein purity was assessed by SDS-PAGE, which showed a single band consistent with the expected molecular weight of *H. pylori* PNP (Figure S1).

**Figure 1:**
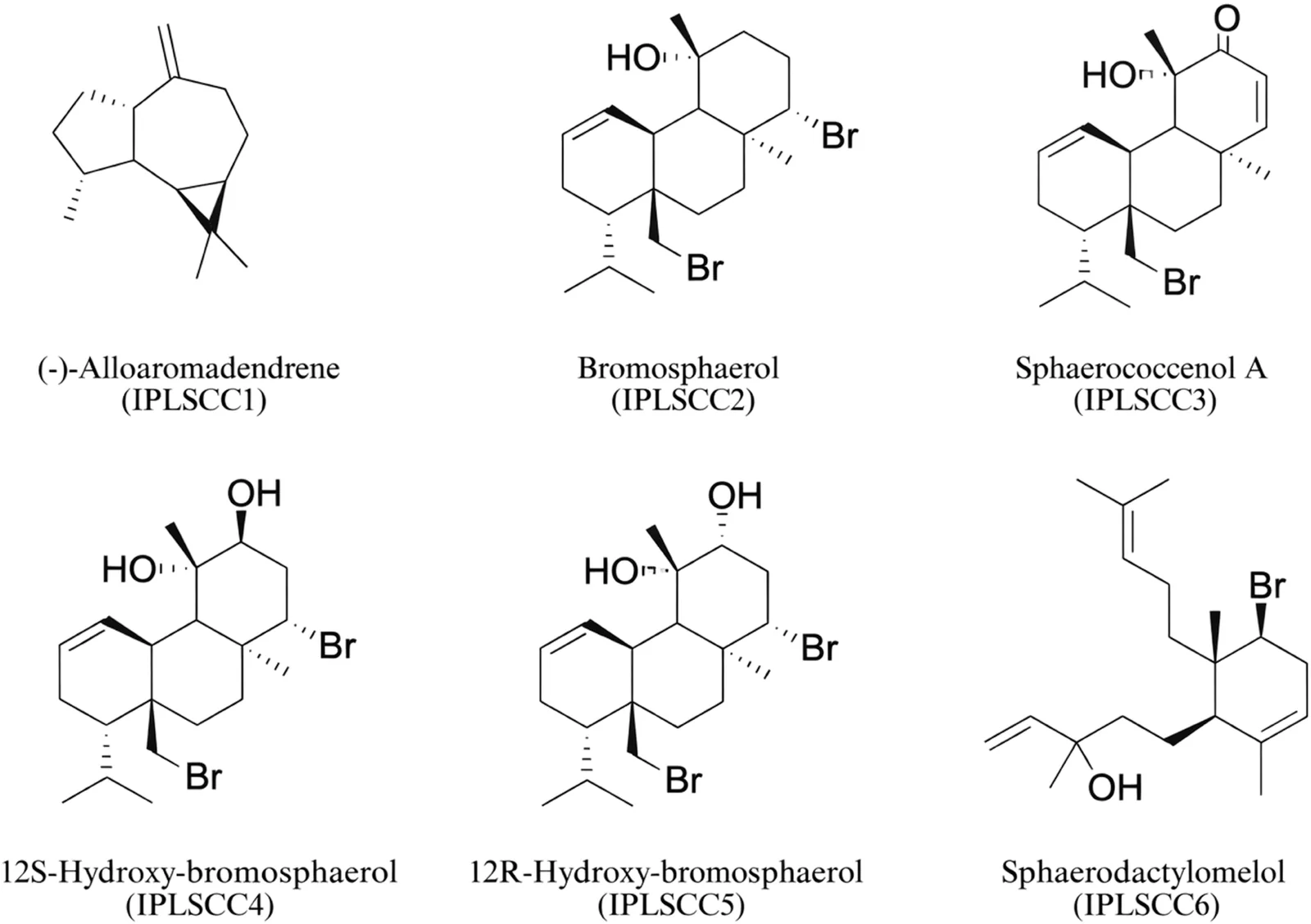
Chemical structures of six terpenoids isolated from *Sphaerococcus coronopifolius*.

The catalytic activity of recombinant *H. pylori* PNP was assessed by steady-state kinetic analysis. Initial reaction velocities were measured as a function of substrate concentration and fitted to the Michaelis–Menten model, resulting in a Michaelis–Menten constant (*K_m_*) value of 40.21 μM (95% CI: 33.10–49.20) for the substrate m^7^Guo (*R* ^2^ = 0.9876) (Figure 2A). This value is consistent with previously reported kinetic parameters for bacterial PNPs, supporting the functional integrity of the recombinant enzyme.^28^

**Figure 2:**
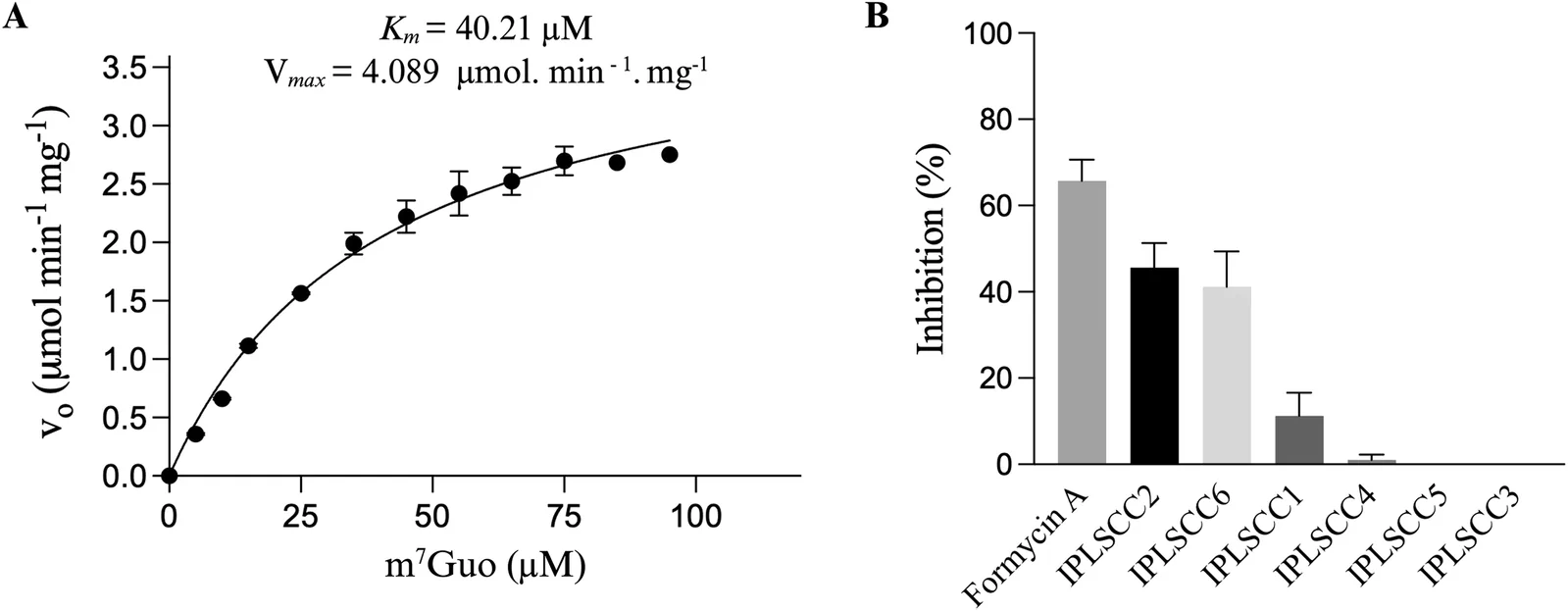
Kinetic characterization and inhibition screening of recombinant *H. pylori* PNP. (A) Steady-state kinetic analysis of recombinant *H. pylori* PNP with a C-terminal His-tag. Initial velocities (*v*_0_) were measured using m^7^Guo concentrations ranging from 0 to 95 μM at a fixed inorganic phosphate concentration of 50 mM. Data points represent mean ± SD of technical triplicate measurements, and the solid line corresponds to nonlinear regression fitting using the Michaelis–Menten equation (*K_m_* = 40.21 μM, 95% CI 33.10–49.20 μM; *V*_max_ = 4.089 μmol·min^–1^·mg^–1^, 95% CI 3.770–4.479 μmol·min^–1^·mg^–1^; *R* ^2^ = 0.9876) performed in GraphPad Prism 8.0.1. (B) Single-point inhibition of *H. pylori* PNP by six terpenoids isolated from *Sphaerococcus coronopifolius* and the reference inhibitor Formycin A, all evaluated at 30 μM. Inhibition (%) was calculated relative to the DMSO-treated control, defined as 0% inhibition. Formycin A was included as a reference inhibitor (64.42 ± 4.93%). Bromosphaerol (IPLSCC2) and sphaerodactylomelol (IPLSCC6) showed reproducible inhibitory activity (46.04 ± 4.70% and 39.39 ± 8.24%, respectively), while the remaining compounds displayed only marginal or no inhibition (IPLSCC1:12.28 ± 5.20%; IPLSCC4:0.53 ± 1.37%; IPLSCC5 and IPLSCC3: no inhibition detected). Data represent mean ± SD of two independent experiments, conducted on different days, each performed in technical triplicate.

The inhibitory potential of six terpenoids isolated from *S. coronopifolius* was initially assessed using a single-concentration screening approach in a direct spectrophotometric assay. The screening concentration of 30 μM was adopted as a single standardized concentration applied to all compounds, including the reference inhibitor formycin A, to allow direct comparison within the same assay. This value represented the highest concentration compatible with the aqueous solubility of the compounds under the assay conditions and with the limited quantities of purified terpenoids available from wild-collected algal biomass, which precluded full concentration–response profiling at this stage.

Enzymatic activity was normalized to a vehicle-treated control (DMSO), defined as 100% activity, while formycin A was included as a positive control, reducing enzyme activity by 64.42 ± 4.93% under the present assay conditions. Formycin A was selected as the reference inhibitor because it is a well-characterized competitive inhibitor of bacterial PNPs, for which a crystal structure in complex with *H. pylori* PNP has been resolved.^28^ Because reported data for formycin A against bacterial PNPs are limited to inhibition constant (*K_i_*) values, which vary across species (∼5 μM for *E. coli* PNP versus ∼14 μM for *H. pylori* PNP),^14,28^ and because *K_i_* reflects binding affinity rather than inhibition at a fixed concentration, the 64.42% inhibition observed here is not directly comparable to those values and should not be interpreted as indicating weak target engagement.

Among the tested compounds, bromosphaerol and sphaerodactylomelol reproducibly reduced *H. pylori* *PNP* activity at 30 μM (46.04 ± 4.70% and 39.39 ± 8.24%, respectively), whereas the remaining terpenoids displayed only marginal or no inhibition under the same conditions (Figure 2B). These results indicate that two out of six compounds isolated from a single algal species showed detectable enzyme-level activity, indicating that *S. coronopifolius*-derived compounds can interact with this bacterial enzyme. The single-concentration screening performed in this study represents an initial assessment of the ability of these compounds to influence *H. pylori* PNP enzymatic activity and provides a basis for further characterization. Among the evaluated compounds, bromosphaerol and sphaerodactylomelol showed the most pronounced effects and were therefore considered for further structural investigation.

To gain structural insight into the putative binding mode of the compounds within the active site of *H. pylori* PNP, molecular docking and molecular dynamics simulations were performed. To ensure reliable docking results, the protocol was validated through a redocking study using crystallographic complexes of *H. pylori* PNP, employing GOLD 2020.3 and evaluating the four available scoring functions (Table S1). Redocking results were classified into six categories based on Root mean square deviation (RMSD) values and the corresponding scoring function outputs (Table 1). When the lowest-energy docked pose corresponded to an RMSD value below 2.0 Å relative to the crystallographic ligand, the docking protocol was considered capable of reproducing experimental binding modes.^29^ Based on this criterion, comparative analysis of the evaluated scoring functions showed that ChemPLP and ASP consistently yielded the highest proportion of low-RMSD solutions, supporting their selection as the primary scoring and rescoring functions, respectively (Table 1).

**Table 1:** Number of *Helicobacter pylori* PNP Structures Classified by the RMSD Values Obtained with Each GOLD Scoring Function

RMSD	0.0–0.99	1.0–1.99	2.0–2.99	3.0–3.99	4.0–4.99	>5.0
ChemPLP	4	0	0	2	0	2
ChemScore	1	0	0	1	0	6
GoldScore	2	0	0	1	2	3
ASP	3	0	1	2	0	2

Because *H. pylori* PNP is a hexameric enzyme whose active sites can adopt different conformations within the same oligomer, available crystallographic structures were further analyzed using CASTp to account for receptor structural variability.^16,28,30^ This analysis enabled quantitative comparison of active-site pocket volumes across individual chains (Table S2), revealing pronounced heterogeneity consistent with previous structural studies.^28^ Among the structures identified, representative conformations were selected based on CASTp-derived pocket volumes (Table S2) in combination with crystallographic quality indicators available from the Protein Data Bank, including resolution and electron density of bound ligands, where applicable. Three receptor conformations were selected for docking analyses: a compact active site corresponding to a closed conformation in the presence of a bound ligand (6F4X, chain C), an open active site in the absence of ligand (6G7X, chain F), and a structure containing a bound ligand displaying the largest pocket volume (6F4W, chain D) (Figure S2).

Docking simulations were subsequently performed for bromosphaerol and sphaerodactylomelol using three crystallographic structures of *H. pylori* PNP (PDB IDs 6F4X, 6G7X, and 6F4W), selected to represent distinct active-site conformational states. The predicted binding poses revealed interaction patterns involving residues of the purine-binding cavity across all three receptor conformations. Hydrophobic contacts were consistently observed with Phe159 and Leu206, which have been previously identified as key components of the base-binding region of the *H. pylori* *PNP* active site,^14,28^ while a hydrogen bond was predicted between the hydroxyl group of bromosphaerol and the backbone carbonyl oxygen of Ile178 (Figure 3A). For sphaerodactylomelol, hydrophobic contacts with the same residues were observed with a hydrogen bond involving the backbone carbonyl of Phe159 (Figure 3B). The conservation of these interactions across the three receptor states supports the reliability of the predicted binding regions.

**Figure 3:**
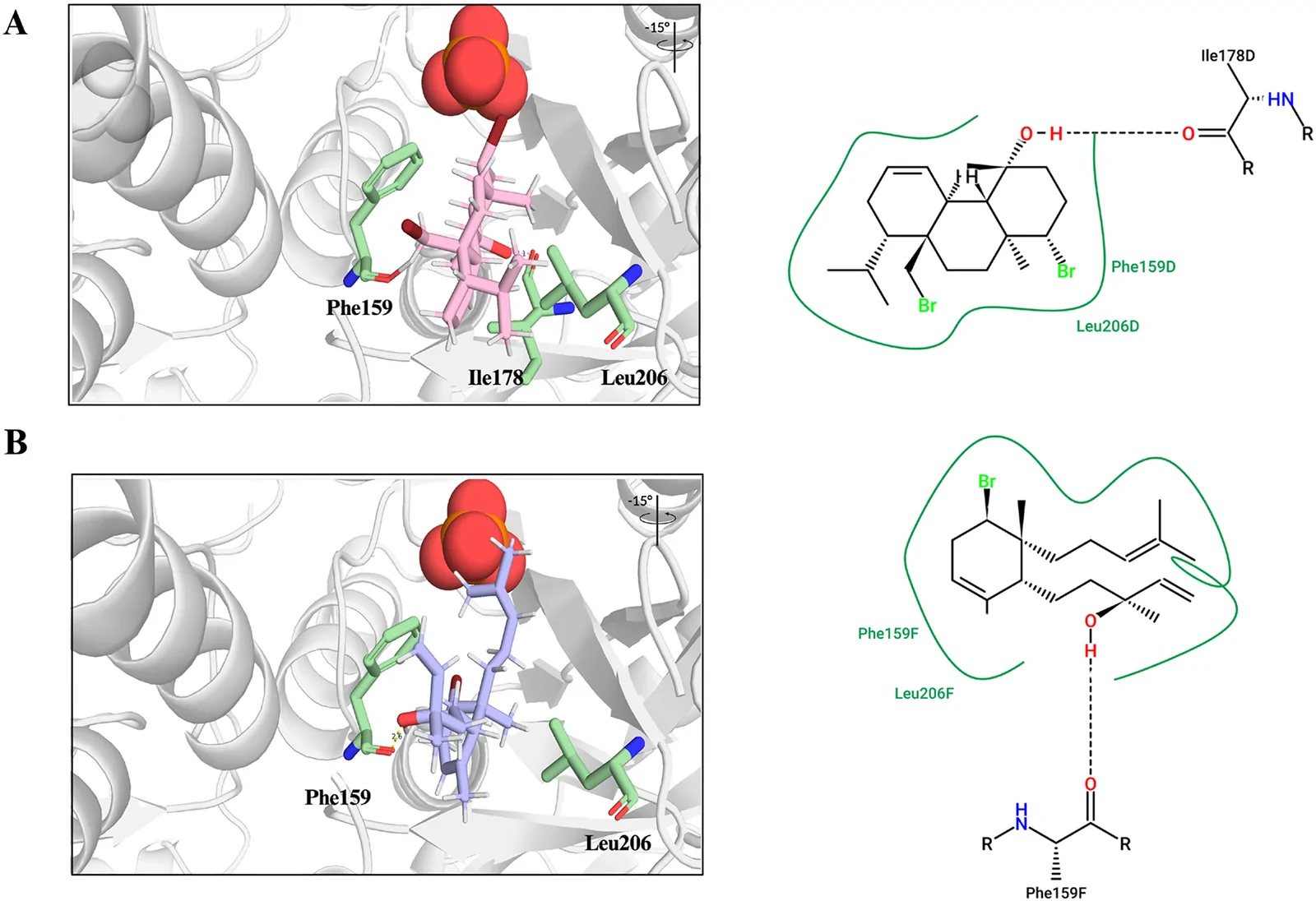
Predicted binding modes of bromosphaerol (A) and sphaerodactylomelol (B) in the active site of *H. pylori* PNP, obtained by molecular docking using GOLD 2020.3. Left panels show the protein–ligand complexes visualized in PyMOL: bromosphaerol (light pink) docked into the open conformation with bound ligand (6F4W, chain D), and sphaerodactylomelol (light blue) docked into the open apo conformation (6G7X, chain F); interacting residues (Phe159, Ile178, and Leu206 in panel A; Phe159 and Leu206 in panel B) are shown in green. Right panels show the corresponding two-dimensional interaction diagrams generated with PoseView, depicting hydrophobic contacts (green curves) and hydrogen bonds (dashed lines) with the backbone carbonyl of Ile178 (A) and Phe159 (B).

However, the bulkier terpenoid scaffolds were better accommodated within the open conformations (6G7X, 491.169 Å^3^; 6F4W, 686.963 Å^3^), where the larger pocket volume allowed greater compound burial within the active-site cavity. In contrast, the closed conformation (6F4X, 109.648 Å^3^) provided a more restricted binding environment, with partial exposure of the terpenoid scaffold at the pocket entrance, consistent with the steric constraints imposed by the compact active-site geometry of this state (Figures S3 and S4). Superposition of the docking poses across the three receptor conformations revealed that the binding geometries obtained for the two open conformations (6G7X and 6F4W) were nearly identical, while the pose in the closed conformation (6F4X) showed a distinct positioning of the terpenoid scaffold, consistent with the steric constraints imposed by the reduced pocket volume of this state (Figures S5 and S6). Inspection of the crystallographic binding mode of formycin A in the *H. pylori* PNP structure (PDB 6F4W) reveals that this purine analogue occupies a deeply buried position within the same active-site region, establishing multiple contacts with catalytic residues.^14,28^ Superposition of the docked terpenoid compounds onto this crystallographic reference shows that, while both compounds partially overlap with the formycin A binding region, their terpenoid scaffolds extend toward a more peripheral portion of the pocket, in contrast to the deeper penetration observed for formycin A (Figure S7). Although a direct comparison between crystallographic and docking-derived poses should be made with caution, this more limited and shifted pocket occupancy predicted for the terpenoid compounds relative to formycin A is structurally consistent with the partial inhibition observed biochemically, and suggests that these scaffolds interact with the purine-binding region through a binding mode geometrically distinct from that of classical purine-mimetic inhibitors.^14,15^

Because molecular docking provides static representations of plausible binding modes, molecular dynamics simulations were subsequently employed to further investigate the dynamic behavior of the protein–ligand complexes.^31^ This approach was designed to evaluate the temporal stability of the predicted binding modes and the persistence of key intermolecular interactions, providing complementary insight beyond static structural models.^32^

Molecular dynamics simulations, performed as three independent replicates per system, supported the global structural integrity of *H. pylori* PNP in all three systems (apo, bromosphaerol-bound, and sphaerodactylomelol-bound). RMSD values were monitored independently for each of the six monomers of the hexameric complex, with all chains reaching equilibrium within the first 20–30 ns and remaining stable throughout the 200 ns production phase (Figure 4), with no evidence of conformational drift. The consistency of RMSD profiles across all monomers and across independent replicates indicates that the observed behavior reflects the hexameric assembly as a whole rather than being driven by individual subunits.

**Figure 4:**
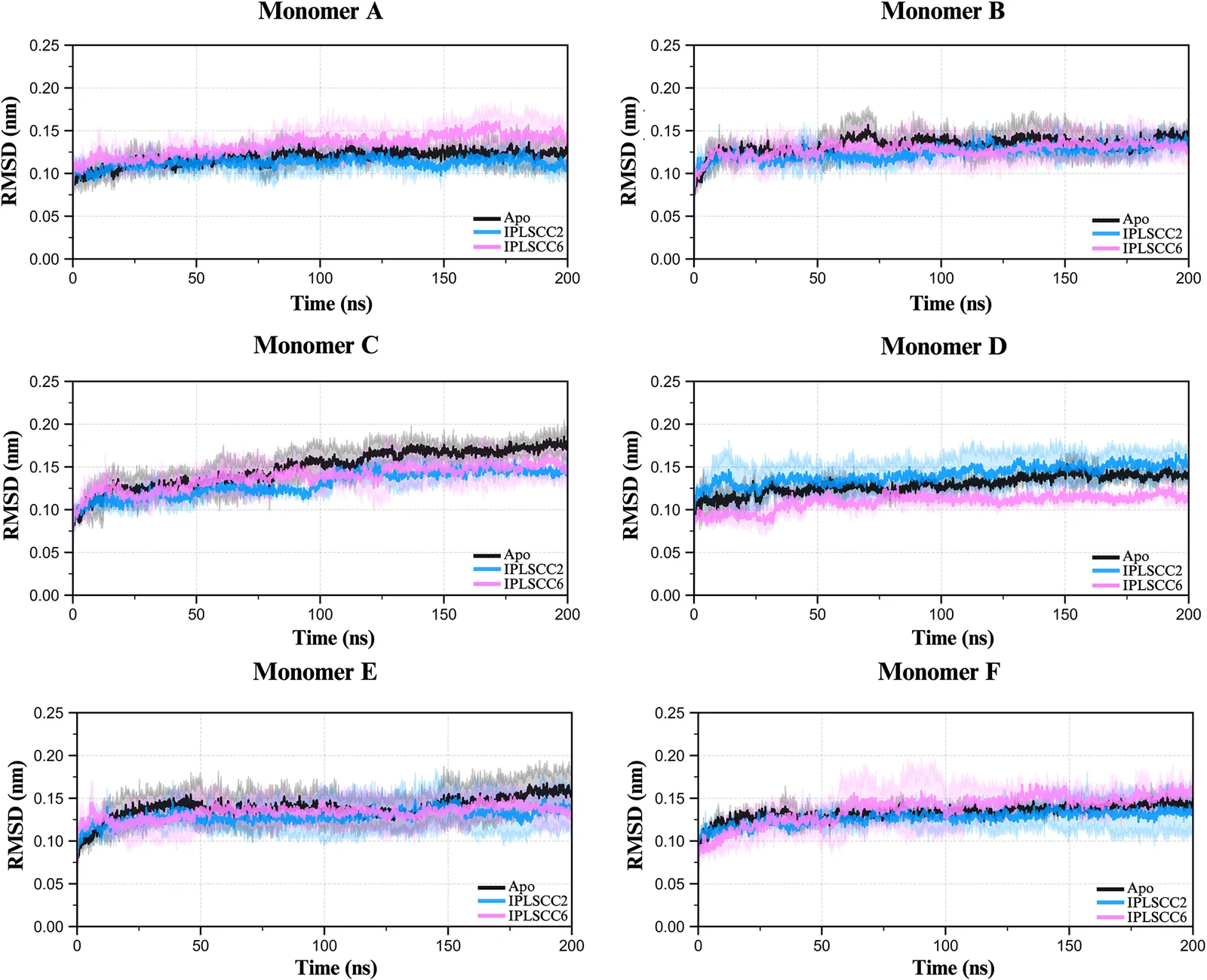
Root mean square deviation (RMSD) of Cα atoms for each monomer (chains A–F) of *H. pylori* PNP during 200 ns molecular dynamics simulations. Results are shown for the ligand-free system (Apo, black), bromosphaerol-bound (IPLSCC2, blue), and sphaerodactylomelol-bound (IPLSCC6, pink) systems. Shaded areas represent the standard deviation for each monomer across three independent simulations.

Per-residue root mean square fluctuation (RMSF) analysis revealed highly similar profiles across the ligand-free, bromosphaerol-bound (IPLSCC2), and sphaerodactylomelol-bound (IPLSCC6) systems throughout the entire sequence (Figure 5), indicating that ligand binding is well tolerated within the active site without perturbing the global structural framework of the enzyme. Taken together, the RMSD and RMSF analyses indicate that the global structure of the protein remained stable throughout the simulations in all three systems, consistent with well equilibrated complexes.

**Figure 5:**
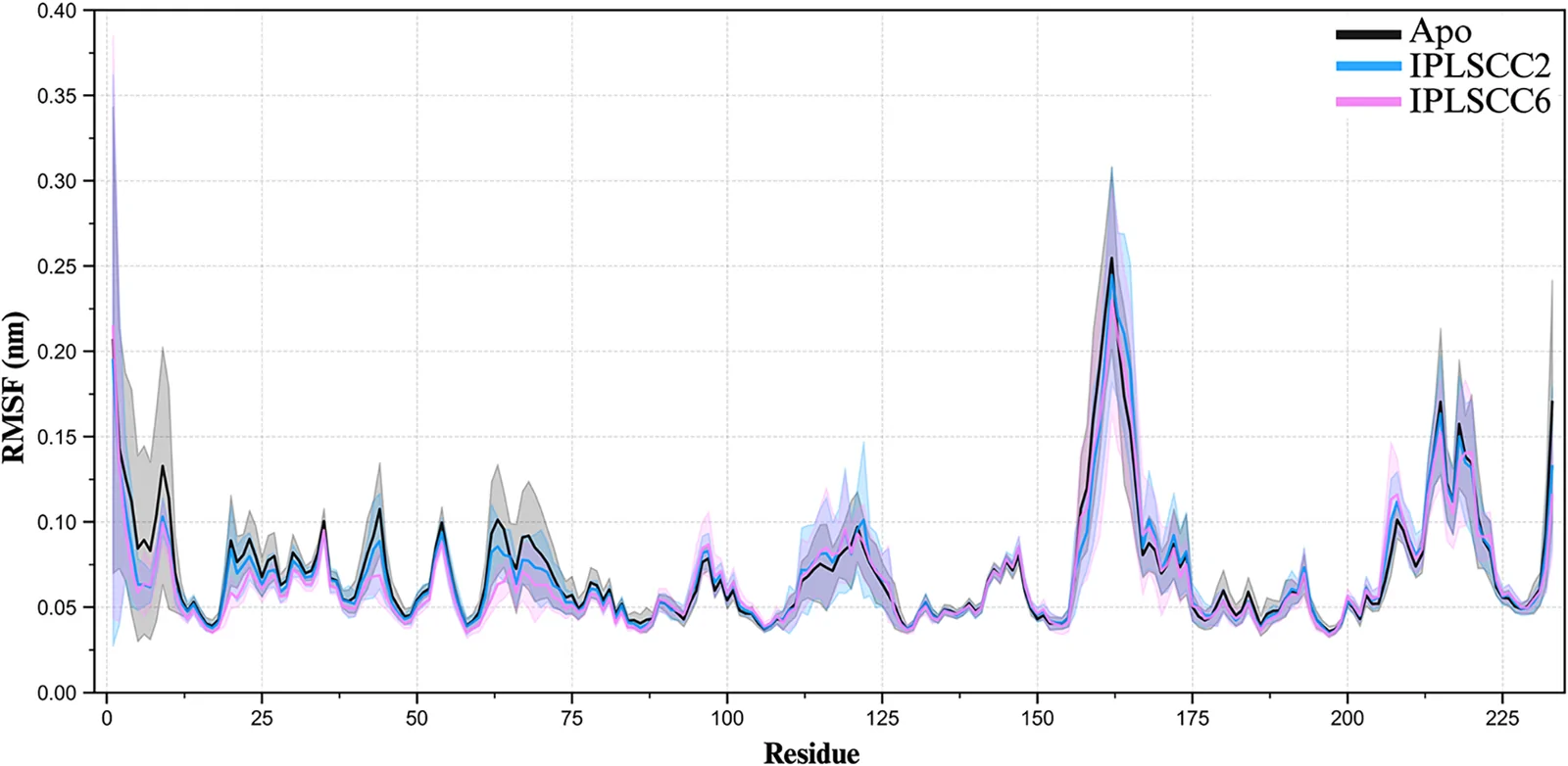
Root mean square fluctuation (RMSF) of Cα atoms per residue of *H. pylori* PNP, averaged across the six monomers of the hexameric complex. Results are shown for the ligand-free system (Apo, black), bromosphaerol-bound (IPLSCC2, blue), and sphaerodactylomelol-bound (IPLSCC6, pink) systems. Shaded areas represent the standard deviation across monomers.

To assess ligand stability within the active site, the RMSD of the heavy atoms of bromosphaerol and sphaerodactylomelol was calculated relative to their initial docked, equilibrated pose across all six monomers independently. Both ligands remained stably bound throughout the 200 ns simulations, with no significant displacement events observed in any chain or replicate (Figure 6). However, the two compounds exhibited markedly different dynamic behaviors within the pocket: bromosphaerol (IPLSCC2) maintained consistently lower RMSD values across all monomers and replicates, indicative of a well anchored binding mode. In contrast, sphaerodactylomelol (IPLSCC6) displayed higher RMSD values and broader variance across replicates, suggesting increased conformational sampling within the active site, despite remaining bound throughout the trajectory. The greater conformational mobility observed for sphaerodactylomelol within the active site may reflect differences in ligand accommodation and binding dynamics compared to those of bromosphaerol.

**Figure 6:**
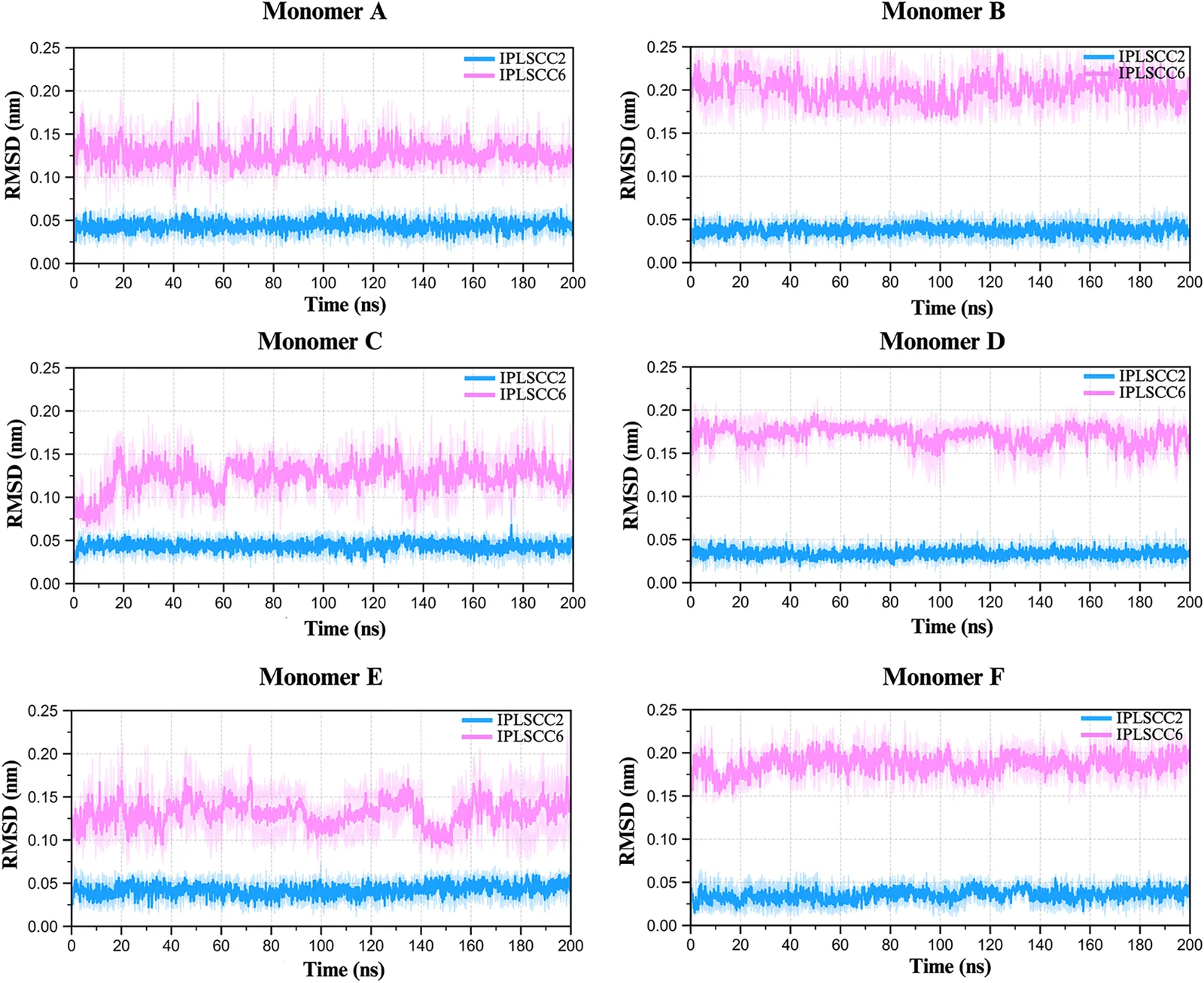
Root mean square deviation (RMSD) of ligand heavy atoms for bromosphaerol (IPLSCC2, blue) and sphaerodactylomelol (IPLSCC6, pink) across the six monomers (chains A–F) of *H. pylori* PNP during 200 ns molecular dynamics simulations. Shaded areas represent standard deviation across the three independent simulations.

The differential dynamic behaviors observed between the two ligands are consistent with their distinct structural features. The base-binding region of the *H. pylori* PNP active site is predominantly hydrophobic, with Phe159 playing a central role in ligand orientation through aromatic stacking interactions, as previously described for nucleoside substrates.^28^ Both bromosphaerol and sphaerodactylomelol are likely retained within this pocket through analogous hydrophobic contacts, particularly involving Phe159 and Leu206, as predicted by molecular docking (Figure 3). Bromosphaerol is a rigid, tricyclic (sphaerene-type) diterpene with limited conformational freedom, which favors a stable orientation within the pocket, as reflected by the lower ligand RMSD values observed across all of the monomers. In contrast, sphaerodactylomelol possesses a monocyclic core with long flexible aliphatic chains and multiple rotatable bonds, allowing it to sample a wider range of poses within the binding site, consistent with the higher ligand RMSD values observed across all of the monomers.

These biochemical and structural findings provide initial evidence that bromosphaerol and sphaerodactylomelol can reduce the activity of *H. pylori* PNP, thereby contributing to the biological characterization of *S. coronopifolius* terpenoids. Previous studies have associated metabolites from this alga with a wide range of biological activities, including cytotoxic and antiproliferative effects,^26,27,33–36^ antimicrobial activity,^22–24,36^ antifouling properties,^37,38^ and antiviral effects.^39^ Despite this diversity of phenotypic outcomes, the molecular targets underlying these activities have remained largely undefined, with existing mechanistic proposals based primarily on computational predictions rather than experimental validation.^27^ Bromosphaerol and sphaerodactylomelol, in particular, have been investigated mainly in cancer-related models,^26,27,33^ as have other diterpenes from this alga.^34,40^ In addition, interactions of bromosphaerol derivatives with the human 20S proteasome have been proposed on the basis of computational analyses.^27^

The previously reported antibacterial activity of these metabolites provides additional context for the present findings. Bromosphaerol and sphaerodactylomelol have shown activity against *Staphylococcus aureus*, with reported MIC values of 22.4 and 96.3 μM, respectively,^23^ and other brominated diterpenes from *S. coronopifolius* have demonstrated activity against multidrug-resistant and methicillin-resistant *S. aureus* strains.^24^ Although these phenotypic effects cannot be attributed to PNP inhibition, and the compounds have not yet been evaluated against *H. pylori* cells, these observations support further investigation of the antibacterial potential of this chemical class.

The biological relevance of purine salvage as a metabolic vulnerability is not restricted to *H. pylori*. In purine-salvage-dependent pathogens such as *Plasmodium* spp. and *Trypanosoma brucei*, disruption of purine acquisition or metabolism can compromise nucleotide availability and pathogen viability.^17,41,42^ Together with the established dependence of *H. pylori* on purine salvage,^13–15^ this precedent provides biological context for investigating *H. pylori* PNP as a target for chemically distinct inhibitors.

A particularly relevant aspect of the present study is the chemical nature of the identified hits. Previously characterized *H. pylori* PNP inhibitors have predominantly consisted of synthetic purine analogues, including 2,6-disubstituted purines and related nucleoside mimetics.^14,15^ In contrast, the marine terpenoids evaluated in the present work represent a structurally distinct class of non-nucleosidic molecules with no structural resemblance to those of classical purine analogues. This distinction is highly relevant, as our findings indicate that these natural compounds display preliminary inhibitory activity against *H. pylori* PNP, suggesting that enzyme interference is not restricted to substrate mimetics. Although further concentration–response analyses are required to determine their precise kinetic parameters and potency, our molecular dynamics simulations provide an important structural context for this interaction. The simulations revealed that both bromosphaerol and sphaerodactylomelol remain stably accommodated within the active-site cavity, supported by persistent hydrophobic contacts particularly involving residues Phe159 and Leu206. These findings are consistent with a binding mode driven by nonspecific hydrophobic contacts within the active-site pocket; this hypothesis derives from the computational analyses and is compatible with, but not established by, the single-concentration biochemical data.

The present findings should therefore be considered an initial hit-identification study. Because the biochemical evaluation was performed at a single compound concentration, concentration–response relationships, inhibitory potency, and mechanism of inhibition remain to be established. Selectivity against human PNP was also not evaluated. Although previously described structural differences between bacterial and human PNPs support the feasibility of selective inhibition,^16,17^ the selectivity of bromosphaerol and sphaerodactylomelol cannot be inferred from the present results and will require direct experimental assessment. Evaluation of these compounds against *H. pylori* cells will likewise be necessary to determine whether inhibition of recombinant *H. pylori* PNP translates into an antibacterial phenotype. Taken together, bromosphaerol and sphaerodactylomelol emerge from this study as preliminary *H. pylori* PNP hits. Rather than establishing these compounds as antibacterial agents, the present findings provide a biochemical and structural basis for their further characterization.

## Conclusion

This study provides initial biochemical evidence that the red macroalga *Sphaerococcus coronopifolius* represents a source of terpenoid scaffolds capable of modulating the activity of *H. pylori* purine nucleoside phosphorylase, a key enzyme in the bacterial purine-salvage pathway. Using a target-based biochemical screening approach combined with molecular docking and molecular dynamics analyses, two of six compounds tested, bromosphaerol and sphaerodactylomelol, were identified as preliminary hits, reducing recombinant *H. pylori* PNP activity by 46.04 ± 4.70% and 39.39 ± 8.24%, respectively, at a screening concentration of 30 μM. Computational analyses predicted stable binding within the purine-binding region of the active site, providing structural models for the putative binding modes of both compounds. The interpretation of these results must consider two primary constraints. Concentration–response characterization and selectivity profiling against the human trimeric homologue were not performed, largely due to the restricted yields of purified metabolites from wild algal biomass. Future experimental validation addressing inhibitory potency and selectivity will be required to confirm the pharmacological relevance of these identified hits. Within these limits, the findings highlight marine macroalgae as an underexplored source of secondary metabolites capable of modulating defined bacterial metabolic targets, and support continued investigation of this chemical space in the search for new antibacterial leads.

## Experimental Section

### General Experimental Procedures

All reagents and solvents used in biochemical experiments were obtained from commercial suppliers and used without further purification unless otherwise stated. Buffer components and salts were of analytical grade. Ultrapure water was obtained using a Milli-Q purification system. All compounds were dissolved in DMSO and visually inspected to ensure complete solubilization under the assay conditions. Compound concentrations were selected to avoid precipitation or turbidity during the enzymatic assays. Steady-state kinetic parameters (*K*_*m*_ and *V*_max_) were determined by nonlinear regression fitting to the Michaelis–Menten equation, and enzyme inhibition data are expressed as mean ± SD of two independent measurements, each performed in technical triplicate. Data analysis and graphical representations were performed using GraphPad Prism 8.0.1 (GraphPad Software, USA). Molecular modeling and visualization were carried out using PyMOL (Schrödinger, LLC). Computational simulations were performed using the software packages specified in the corresponding sections.

### Compound Extraction and Isolation

Six terpenoids, including five brominated diterpenes, were obtained from the red macroalga *Sphaerococcus coronopifolius* Stackhouse, 1797, collected in the Berlenga Nature Reserve, Peniche, Portugal (Flandres Bay; 39°24′44.8″N, 9°30′29.5″W), under prior authorization of ICNF–Instituto da Conservação da Natureza e das Florestas. Samples were collected by scuba diving at depths of 10–14 m and transported to the MARE-IPLeiria facilities, where they were washed with seawater to remove detritus, epiphytes, and encrusting material. The cleaned algal biomass was freeze-dried, ground, and sequentially extracted with methanol and dichloromethane. The dichloromethane extract was further fractionated by vacuum liquid chromatography on silica gel, followed by successive purification steps using silica gel column chromatography and semipreparative reversed-phase HPLC, yielding alloaromadendrene, sphaerodactylomelol, bromosphaerol, sphaerococcenol A, 12S-hydroxy-bromosphaerol, and 12R-hydroxy-bromosphaerol. Structural elucidation was achieved by extensive spectroscopic analysis, including NMR and MS, and comparison with literature data. The compounds were kindly provided by collaborators, and the collection, extraction, isolation, purification, and structural characterization procedures were performed as previously described.^23,33^ All compounds were dissolved in DMSO prior to biochemical assays. Full experimental details of isolation and identification are provided as Supporting Information.

### Protein Expression and Purification

A pET-22a­(+) expression vector containing the *Helicobacter pylori* *deoD* gene and a C-terminal His-tag was acquired from GenOne (Brazil). Recombinant protein expression was initially attempted using conditions previously reported for *Escherichia coli* PNP (BL21­(DE3), 37 °C, induction at OD_600_= 0.6 with 0.2 mM IPTG for 4 h);^43^ however, no detectable expression of recombinant *H. pylori* PNP was obtained under these conditions. Therefore, the expression protocol was empirically adapted by replacing *E. coli* BL21­(DE3) with BL21­(DE3)­pLysS, increasing the IPTG concentration from 0.2 to 0.5 mM, and extending the induction period from 4 to 15 h. The induction temperature (37 °C) and OD_600_ (0.4–0.6) were maintained. Under the conditions tested, this modified protocol resulted in detectable production of soluble recombinant protein (Figure S1). *E. coli* BL21­(DE3)­pLysS cells were transformed with the recombinant pET-22a­(+) vector by heat shock and selected in Luria–Bertani (LB) medium supplemented with 50 μg/mL ampicillin and 34 μg/mL chloramphenicol. A single colony was inoculated into 30 mL of LB medium containing the same concentrations of both antibiotics and cultured overnight at 37 °C and 180 rpm. The starter culture was then transferred to 1 L of LB medium supplemented with the same antibiotics and grown at 37 °C and 180 rpm. When the culture reached an OD_600_ of 0.4–0.6, recombinant protein expression was induced with 0.5 mM isopropyl β-D-1-thiogalactopyranoside (IPTG). The culture was incubated for an additional 15 h at 37 °C and 180 rpm, after which the cells were harvested by centrifugation at 2130*g* for 20 min at 4 °C. The cell pellet was resuspended in lysis buffer containing 50 mM HEPES–NaOH (pH 6.8), 200 mM NaCl, 2 U/mL DNase, 0.2 mg/mL lysozyme, and protease inhibitor cocktail and incubated on ice for 30 min. Cells were disrupted by four 20 s sonication cycles, with 1 min intervals between cycles. The lysate was centrifuged at 13,300*g* for 1 h at 4 °C to separate the soluble and insoluble fractions. Recombinant *H. pylori* PNP was purified from the soluble fraction by immobilized metal affinity chromatography using a 5 mL Ni-NTA column. The column was equilibrated with 50 mM HEPES–NaOH (pH 6.8) and 100 mM NaCl, and bound proteins were eluted using a 25–500 mM imidazole gradient. Purification was performed at room temperature, and the collected fractions were analyzed by SDS-PAGE (Figure S1). Following purification, imidazole was removed by buffer exchange using an Amicon Ultra centrifugal filter (30 kDa MWCO; Millipore). The protein was first washed with buffer containing 100 mM NaCl and 1 mM EDTA and subsequently exchanged into 50 mM HEPES–NaOH (pH 6.8) to prevent protein precipitation. The purified protein was concentrated using the same centrifugal device. The final protein concentration was determined using the Bradford assay, with bovine serum albumin (BSA; 0.1–1.4 mg/mL) as the standard. Absorbance was measured at 595 nm using a SpectraMax i3 microplate reader (Molecular Devices, LLC, USA).

### Kinetic Characterization of *Helicobacter pylori* PNP Enzyme

*H. pylori* PNP activity was determined by a direct spectrophotometric method based on the absorbance change resulting from the conversion of the substrate 7-methylguanosine (m^7^Guo) into free purine base in the presence of inorganic phosphate, using a differential molar extinction coefficient of Δε260 = 4600 M^–1^·cm-1 ^44,45^ and a 1 cm path length to convert absorbance changes into reaction velocity, according to *v*_0_ (μmol·min^–1^·mg^–1^) = (ΔAbs·min^–1^ × 10^6^)/(|Δε| × [enzyme]­μM × MW_kDa), where [enzyme] is the molar concentration of enzyme used in the reaction (0.2 μM) and MW is the monomer molecular weight of *H. pylori* PNP (26.85 kDa), calculated from the amino acid sequence of the His-tagged construct using ProtParam (ExPASy).^46^ Measurements were performed at 25 °C using a UV-2550 spectrophotometer (Shimadzu), with absorbance monitored at 260 nm. Initially, the Michaelis–Menten constant (*K_m_*) for m^7^Guo was determined under saturating inorganic phosphate conditions (50 mM).^28^ The enzyme concentration was adjusted to 0.2 μM to ensure a linear response over the reaction time. Kinetic experiments were performed in technical triplicate. The reaction mixture contained 50 mM HEPES-NaOH buffer pH 6.8, 50 mM phosphate buffer pH 6.8, varying concentrations of m^7^Guo, and the purified enzyme. The reaction was initiated by the addition of the enzyme to the mixture, and the change in absorbance was monitored for 60 s. For each substrate concentration assayed, the velocity of the reaction was calculated based on the initial absorbance change over 60 s. The resulting reaction velocities were plotted against their respective substrate concentrations, and the kinetic parameter (*K_m_*) was determined by nonlinear regression fitting to the Michaelis–Menten equation using GraphPad Prism 8.0.1 (GraphPad Software, USA). A substrate concentration of 40 μM, closely approximating the *K_m_* value obtained (40.21 μM), was subsequently used as the fixed m^7^Guo concentration in the inhibition assays described below.

### *Helicobacter pylori* PNP Enzyme Inhibition Assays

To evaluate the inhibitory potential of selected compounds against *H. pylori* PNP, enzymatic assays were performed under the same conditions established during the kinetic characterization. The concentration of the substrate m^7^Guo was fixed at the previously determined *K_m_* value, while inorganic phosphate was maintained at a saturating concentration (50 mM). All reactions were carried out in technical triplicate, in two independent experiments. Each compound was tested at a final concentration of 30 μM. The reaction mixtures contained 50 mM HEPES-NaOH buffer pH 6.8, 50 mM phosphate buffer pH 6.8, purified *H. pylori* PNP, and the test compound. Initially, the reaction mixtures were incubated for 5 min at 25 °C. The reactions were then initiated by the addition of m^7^Guo at its *K_m_* concentration and monitored by measuring absorbance at 260 nm for 1 min at 25 °C using a UV-2550 spectrophotometer (Shimadzu). For all reactions, the volume of DMSO was adjusted to a final concentration of 0.6%. Formycin A was used as a positive control for inhibition. Relative enzyme activity (%) was calculated by considering the activity in the absence of inhibitors as 100%. The inhibitory effect of each compound was determined by comparing the reaction rate in the presence and absence of the compound. Compound-only control reactions (without enzyme) were performed under the same assay conditions to assess potential spectral interference at 260 nm. None of the six compounds tested showed significant absorbance or a measurable slope at 260 nm over 60 s in the absence of enzyme.

### Redocking

To validate the molecular docking protocol, a redocking study was performed using eight crystallographic structures of *H. pylori* PNP in complex with ligands, selected from the Protein Data Bank (PDB) with resolutions ≤2.5 Å (Table S1). The redocking strategy and evaluation criteria were defined based on previously established docking protocols for enzyme–ligand systems and adapted for the present study.^47^ All structures were structurally aligned to the reference structure PDB ID: 5LU0. Prior to docking, proteins were prepared by removing water molecules, bound ligands, and crystallographic artifacts. Hydrogen atoms were added using GOLD 2020.3.^48^ Docking simulations were performed in GOLD using a genetic algorithm with a search efficiency setting of 200% (Slow), employing four scoring functions: ChemPLP, GoldScore, ChemScore, and ASP. The binding site was defined as a spherical region (10 Å radius) centered on a reference atom located at the geometric center of a cocrystallized ligand within the active site. RMSD values between the docked poses and their corresponding crystallographic ligands were used to assess protocol accuracy, and results were categorized into six RMSD classes.

### Conformational Analysis of PNP for Molecular Docking

*H. pylori* PNP is a hexameric enzyme with six active sites, which can adopt both open and closed conformations within the same structure.^16,28^ To ensure the selection of biologically relevant models for molecular docking, all crystallographic PNP structures available in the Protein Data Bank (PDB), including both apo- and ligand-bound forms, were analyzed using CASTp 3.0.^30^ This analysis involved the calculation of solvent-accessible volumes of the active-site pockets to identify structural models with different conformational states and assess potential steric restrictions. On the basis of this evaluation, three representative structures with distinct active-site conformations were selected for subsequent docking studies.

### Molecular Docking

The two compounds with the highest inhibitory activity against *H. pylori* PNP were selected for molecular docking simulations to investigate their potential interactions with the enzyme. Three protein structures representing distinct active-site conformations (a closed conformation with a compact active site, an open ligand-bound conformation with the largest pocket volume, and an open apo conformation) were selected based on prior conformational analysis. Protein structures were prepared in GOLD 2020.3 by removing water molecules and cofactors and adding hydrogen atoms.^48^ Ligand structures were also prepared using the same software. For each compound, 20 docking poses were generated using a genetic algorithm with a search efficiency setting of 200% (Slow). The binding site was defined as a spherical region of 12 Å radius centered on a reference atom (*x*: 0.908, *y*: 32.312, *z*: 20.200) located within the active site. Docking simulations were performed using ChemPLP as the primary scoring function and ASP for rescoring, as these functions provided a higher number of docked poses with RMSD ≤ 2.0 Å in the redocking validation. The final docking pose for each compound was selected based on the highest ChemPLP score, confirmed by consensus with the ASP rescoring. Crystallographic water molecules were removed prior to docking, as no conserved water molecules were identified mediating key interactions within the *H. pylori* PNP active site, and their removal is standard practice to allow full exploration of the binding cavity by the bulkier terpenoid structures evaluated in this study. The resulting protein–ligand complexes were visualized using PyMOL (The PyMOL Molecular Graphics System, Schrödinger, LLC), and their molecular interactions were analyzed using PoseView.

### Molecular Dynamics Simulations

Molecular dynamics (MD) simulations were performed for bromosphaerol and sphaerodactylomelol in complex with the enzyme, as well as for the ligand-free (apo) form, to evaluate the stability of the docked poses and the behavior of the unliganded enzyme. Three independent simulations were performed for each system (apo, bromosphaerol-bound, and sphaerodactylomelol-bound), totaling nine 200 ns simulations. Both apo and holo forms of the enzyme included inorganic orthophosphate as a cosubstrate in the active site, consistent with the catalytic mechanism of purine nucleoside phosphorylase. Simulations were carried out using the GROMACS software package (version 2022.2)^49^ with the Amber99SB-ILDN force field.^50^ Ligand parameters were generated using the ACPYPE interface^51^ based on the General Amber Force Field (GAFF).^52^ The protein–ligand complexes were solvated in a cubic box filled with TIP3P water molecules^53^ extending 10 Å from the protein surface. Periodic boundary conditions were applied in all directions, and counterions were added to neutralize the system. Energy minimization was performed using the steepest-descent algorithm until a tolerance of 100 kJ·mol^–1^·nm^–1^ was reached. The system was then equilibrated for 1 ns under constant volume and temperature (NVT), followed by 1 ns under constant pressure and temperature (NPT). Temperature was controlled using the V-rescale thermostat,^54^ and pressure was maintained at 1 bar using the Parrinello–Rahman barostat.^55^ All covalent bonds involving hydrogen atoms were constrained using the LINCS algorithm,^56^ allowing the use of a 2 fs integration time step. Electrostatic interactions were calculated using the particle mesh Ewald (PME) method^57^ with a real-space cutoff of 1.0 nm. A reciprocal grid of 80 × 80 × 80 cells with fourth-order B-spline interpolation was used. van der Waals interactions were calculated using a cutoff of 1.025 nm, and neighbor lists were updated every 20 steps. Production MD simulations were performed for 200 ns per replicate, with three independent replicates for each system. Trajectory analysis was carried out using built-in GROMACS tools to calculate RMSD and RMSF. Structural visualization was performed using PyMOL.

## Supplementary Material



## Data Availability

The configuration file for molecular dynamic simulations, along with the PDB files of the protein–ligand complexes, is available at no cost at the following link: https://github.com/luisfernandosaraiva/Molecular_dynamics_Macroalgae

## Notes

Isolation and identification of compounds (Section S1); RMSD values of redocking with the four GOLD scoring functions (Table S1); CASTp active-site pocket volumes of selected *H. pylori* PNP crystallographic chains (Table S2); SDS-PAGE of His-tagged *H. pylori* PNP purification (Figure S1); molecular surface representations of the three receptor conformations selected for docking (Figure S2); docking poses of bromosphaerol (Figure S3) and sphaerodactylomelol (Figure S4) across three receptor conformations in molecular surface representation; docking poses of bromosphaerol (Figure S5) and sphaerodactylomelol (Figure S6) using three receptor structures in cartoon representation; comparison of the docking poses of bromosphaerol and sphaerodactylomelol with crystallographic formycin A in the 6F4W structure (Figure S7) (PDF)
