# Supplementary material for: Secondary Metabolites from the Red Macroalga Sphaerococcus coronopifolius as Hit Candidates of Helicobacter pylori Purine Nucleoside Phosphorylase
Source: ACS Omega. 2026 Oct 8;11(41):62973–84. doi: 10.1021/acsomega.6c05168 (PMC13688487; doi:10.1021/acsomega.6c05168)

**Secondary Metabolites from the Red Macroalga *Sphaerococcus coronopifolius* as Hit Candidates of *Helicobacter pylori* Purine Nucleoside Phosphorylase**

Ana Micaela Camini[1,2], Maria Eduarda Delawi[1,2], Bruno Rampanelli Dahmer[1,2], Manuella Silva da Costa[1,2], Celso Miguel da Maia Alves[3], Joana Rita Martins da Silva[3], Rui Filipe Pinto Pedrosa[3], Jeferson Camargo de Lima[1,2,4], Luís Fernando Saraiva Macedo Timmers[1,2,4*]

[1]Universidade do Vale do Taquari, Univates - Lajeado, Rio Grande do Sul, Brazil

[2]Programa de Pós-Graduação em Biotecnologia (PPGBiotec), Univates - Lajeado, Rio Grande do Sul, Brazil

[3]MARE—Marine and Environmental Sciences Centre, ARNET—Aquatic Research Network, Politécnico de Leiria, 2520-630 Peniche, Portugal

[4]Programa de Pós-Graduação em Ciências Médicas (PPGCM), Univates - Lajeado, Rio Grande do Sul, Brazil

*Corresponding Author: e-mail: luis.timmers@univates.br

## Section S1. Isolation and Identification of Compounds

### Collection and Extraction

Fresh specimens of *Sphaerococcus coronopifolius* were harvested at Berlenga Nature Reserve, Peniche, Portugal (Flandres Bay; 39°24′44.8″N, 9°30′29.5″W), under prior authorization from ICNF – Instituto da Conservação da Natureza e das Florestas. Algal material was collected by SCUBA diving at depths ranging from 10 to 14 m and promptly transferred to MARE-IPLeiria facilities. Upon arrival, specimens were rinsed thoroughly with seawater to eliminate detritus, epiphytes, and encrusting organisms. The clean biomass (19.180 kg) was freeze-dried (Scanvac Cool Safe, LaboGene, Lynge, Denmark), after which the dried material was ground and kept at room temperature until further use.

A portion of the freeze-dried material (2.777 kg) was subjected to sequential solvent extraction using methanol (MeOH, Fisher Scientific, UK) followed by dichloromethane ($CH_2Cl_2$, VWR, France), at a solvent-to-biomass ratio of 40:1 (v/w), under continuous mechanical stirring at room temperature for 12 h. The $CH_2Cl_2$ extract was concentrated under reduced pressure using a rotary evaporator, yielding 23.637 g of dry residue.

### Fractionation by Vacuum Liquid Chromatography

The concentrated extract was fractionated by normal-phase vacuum liquid chromatography (VLC) on silica gel 60 (0.06–0.20 mm; Scharlau, Spain). Elution was carried out with cyclohexane (VWR, France) containing stepwise increasing proportions of ethyl acetate (EtOAc, VWR, Belgium) in 25% increments (400 mL per step), affording five fractions: F1 (2.779 g), F2 (6.006 g), F3 (4.115 g), F4 (0.595 g), and F5 (0.326 g). All fractions were concentrated to dryness under reduced pressure.

### Isolation of Individual Compounds

*Fraction F1* was further purified over a silica gel 60 column (column dimensions: 25 cm height × 4 cm diameter; collection volume: 10 mL per fraction) using a gradient mobile phase of *n*-hexane (*n*-Hex, Panreac, AppliChem, Germany) and EtOAc of increasing polarity. Elution from 100% *n*-Hex through *n*-Hex/EtOAc (1:1) provided 291 fractions (f1–f291), followed by *n*-Hex/EtOAc (3:7) yielding 18 additional fractions (f292–f307); the column was subsequently washed with 100% EtOAc and 100% MeOH (500 mL each). Fractions f5–f7 were combined and concentrated to yield alloaromadendrene (220.8 mg).

*Fractions F2 and F3* were combined and subjected to semi-preparative reversed-phase HPLC (Synergi Fusion-RP 80 Å, Phenomenex, 10 × 250 mm, 4 µm) at 25 °C with a flow rate of 5.19 mL/min. The mobile phase consisted of $H_2O$ and $CH_3CN$ (acetonitrile; Fisher Scientific, UK), programmed as follows: isocratic $H_2O$:$CH_3CN$ 25:75 from 0 to 5 min, a linear gradient to 15:85 over 5–25 min, and isocratic 15:85 thereafter. This separation yielded 12 sub-fractions (P1–P12). Direct isolation from this step afforded sphaerodactylomelol (P5; *t*R 20.0 min; 263.2 mg) and bromosphaerol (P7; *t*R 23.4 min; 267.8 mg).

*Sub-fraction P1* was purified on a silica gel 60 column (Scharlau, 0.04–0.06 mm; 19.5 cm × 2 cm; 10 mL collection volume) eluted isocratically with EtOAc/*n*-Hex (1:9), affording sphaerococcenol A (P1.1; fractions f12–f15; 597.9 mg) and 12*S*-hydroxy-bromosphaerol (P1.2; fractions f34–f60; 101.2 mg).

*Sub-fraction P2* was re-injected onto the semi-preparative reversed-phase HPLC column (Synergi Fusion-RP 80 Å, Phenomenex, 10 × 250 mm, 4 µm) under isocratic conditions ($CH_3CN$/$H_2O$, 60:40) at 5.0 mL/min and 25 °C, affording 7 sub-fractions (P2.1–P2.7). Sub-fraction P2.5 was applied to a silica gel 60 preparative column (Scharlau, 0.04–0.06 mm; 19.0 cm × 2 cm; 10 mL collection volume), eluted with *n*-Hex/EtOAc (8:2) for the first 20 fractions, followed by *n*-Hex/EtOAc (3:7). This step furnished 12*R*-hydroxy-bromosphaerol (fractions f5–f9; 118.1 mg).

**Structural Elucidation**

The structures of all isolated compounds were established by comprehensive spectroscopic analysis, encompassing NMR spectroscopy and mass spectrometry (MS), in conjunction with comparison to previously reported data in the literature.

**Table S1**. RMSD values obtained for *Helicobacter pylori* PNP structures in redocking with the four GOLD score functions.

| PDB ID | RMSD ChemPLP | RMSD ChemScore | RMSD GoldScore | RMSD ASP |
|---|---|---|---|---|
| 5MX4 | 0.5163 | 3.0682 | 6.0604 | 3.0357 |
| 5MX6 | 3.3652 | 8.2379 | 4.8546 | 3.1892 |
| 6F4W | 0.5033 | 0.5416 | 0.7457 | 0.4512 |
| 6F4X | 0.6889 | 5.7124 | 0.5625 | 0.8510 |
| 7OOY | 7.4155 | 7.6943 | 7.3663 | 6.5572 |
| 7OPA | 6.9090 | 6.8365 | 6.8654 | 6.8448 |
| 7OOZ | 0.5009 | 8.0014 | 3.5217 | 0.8466 |
| 7OP9 | 3.0014 | 5.4547 | 4.4025 | 2.4404 |

**Table S2.** Active-site pocket volumes calculated using CASTp for selected crystallographic chains of *H. pylori* PNP[a]. Structures selected for docking calculations are highlighted with boxes.

| PDB | Chain | Active-site volume (Å³) | Active site conformation |
|---|---|---|---|
| 5LU0 | B | 88.050 | Closed |
| 7OOY | B | 107.605 | Closed |
| 5MX4 | C | 109.620 | Closed |
| 6F4X | C | 109.648 | Closed |
| 5MX6 | C | 162.014 | Closed |
| 6F5I | A | 212.754 | Closed |
| 6F4X | E | 216.711 | Closed |
| 5LU0 | C | 219.797 | Closed |
| 7OOZ | B | 243.232 | Closed |
| 5LU0 | A | 275.086 | Closed |
| 6F5I | B | 298.749 | Closed |
| 6F5A | B | 306.580 | Open |
| 5MX6 | E | 341.496 | Open |
| 5LU0 | E | 350.492 | Open |
| 6F4X | F | 362.234 | Open |
| 6F5I | E | 371.357 | Open |
| 6F5I | F | 386.219 | Open |
| 6F4X | A | 395.469 | Open |
| 6F4X | B | 415.256 | Open |
| 6G7X | E | 428.405 | Open |
| 6F5A | C | 454.363 | Open |
| 5MX4 | B | 456.309 | Open |
| 5MX4 | F | 465.008 | Open |
| 6F5A | F | 470.768 | Open |
| 5MX6 | B | 473.333 | Open |
| 5LU0 | D | 479.934 | Open |
| 6F5I | C | 486.782 | Open |
| 6G7X | F | 491.169 | Open |
| 6F5I | D | 503.877 | Open |

| | | | |
|---|---|---|---|
| 6F4X | D | 538.144 | Open |
| 5MX4 | D | 541.030 | Open |
| 6F5A | D | 545.866 | Open |
| 6F4W | B | 553.902 | Open |
| 6F52 | A | 568.857 | Open |
| 6F4W | C | 581.720 | Open |
| 6F4W | A | 583.931 | Open |
| 6F4W | E | 587.146 | Open |
| 6G7X | A | 609.846 | Open |
| 6F4W | F | 609.908 | Open |
| 5MX6 | A | 615.925 | Open |
| 7OOZ | A | 618.200 | Open |
| 6F5A | A | 621.365 | Open |
| 5MX6 | F | 643.533 | Open |
| 6F52 | C | 660.659 | Open |
| 6G7X | B | 668.548 | Open |
| 7OOY | A | 670.906 | Open |
| 5MX6 | D | 674.639 | Open |
| 5LU0 | F | 681.147 | Open |
| 6F4W | D | 686.963 | Open |
| 6F52 | B | 711.545 | Open |
| 5MX4 | E | 712.623 | Open |
| 6G7X | C | 718.881 | Open |
| 5MX4 | A | 729.155 | Open |
| 6F52 | D | 730.823 | Open |
| 6F5A | E | 803.493 | Open |
| 6F52 | E | 839.108 | Open |
| 6G7X | D | 840.153 | Open |
| 6F52 | F | 846.133 | Open |

[a]Pocket volumes were estimated based on solvent-accessible surface calculations using the CASTp server. The classification of the active site as open or closed was determined by visual inspection of the protein surface in PyMOL, with each structure analyzed individually. The assignment was based on the accessibility of the catalytic pocket and on structural descriptions of open and closed conformations previously reported for *H. pylori* PNP (Štefanić et al., 2017; Narczyk et al., 2018).

**Figure S1**. SDS-PAGE of His-tagged *H. pylori* PNP purification. The recombinant protein was purified by immobilized metal affinity chromatography on a Ni-NTA column, with elution using a stepwise imidazole gradient. Molecular weight marker values are indicated in kDa. (A) Lane 1, protein marker; lane 2, insoluble fraction; lane 3, soluble fraction; lane 4, flow-through; lane 5, wash fraction; lanes 6–9, fractions eluted with 25, 75, 100, and 150 mM imidazole, respectively. (B) Lane 1, protein marker; lanes 2–8, fractions eluted with 200, 250, 300, 350, 400, 450, and 500 mM imidazole, respectively. The arrow indicates the expected molecular weight of the His-tagged H. pylori PNP monomer (26.85 kDa). Fractions eluted at 300, 350, and 400 mM imidazole (lanes 4–6 in panel B) were pooled, buffer-exchanged, and concentrated using an Amicon Ultra centrifugal filter (30 kDa MWCO) prior to use in subsequent biochemical assays.

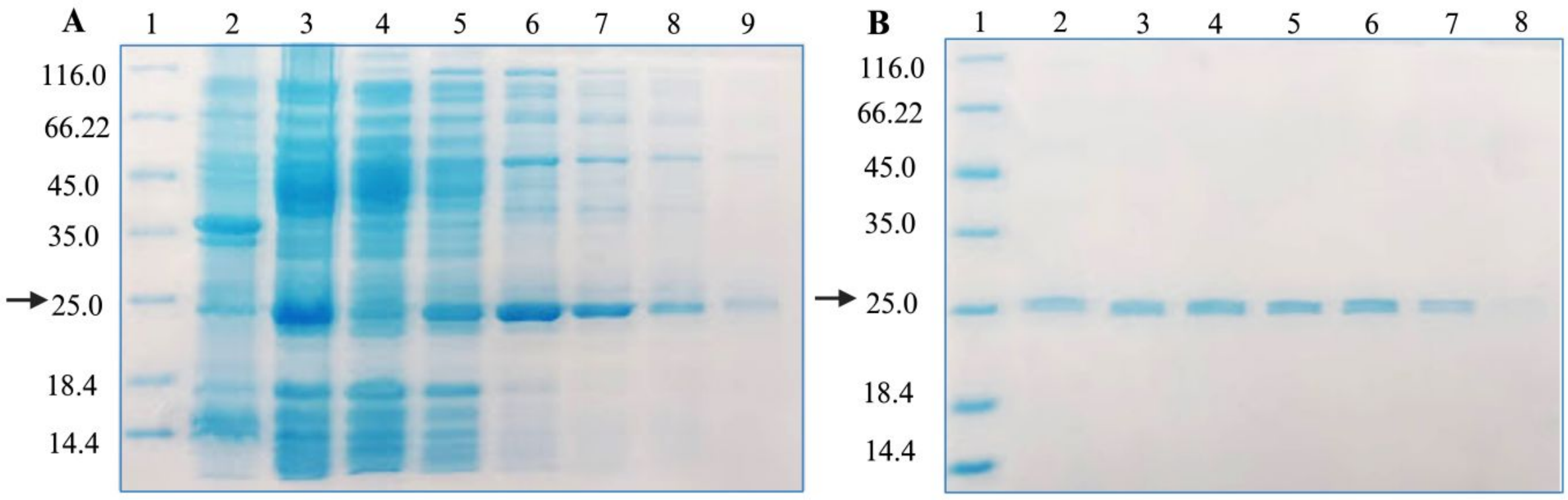

**Figure S2.** Molecular surface representations of the three *H. pylori* PNP receptor conformations selected for molecular docking, illustrating the variation in active-site pocket volume across crystallographic structures. Active-site residues described in the literature are highlighted in pink. **(A)** Closed conformation with compact active site in the presence of a bound ligand (6F4X, chain C; pocket volume = 109.648 Å$^3$); **(B)** open conformation with bound ligand and largest pocket volume (6F4W, chain D; pocket volume = 686.963 Å$^3$); **(C)** open apo conformation in the absence of ligand (6G7X, chain F; pocket volume = 491.169 Å$^3$). The pronounced heterogeneity in pocket volume across conformational states is consistent with the known structural flexibility of the *H. pylori* PNP active site (Narczyk et al., 2018).

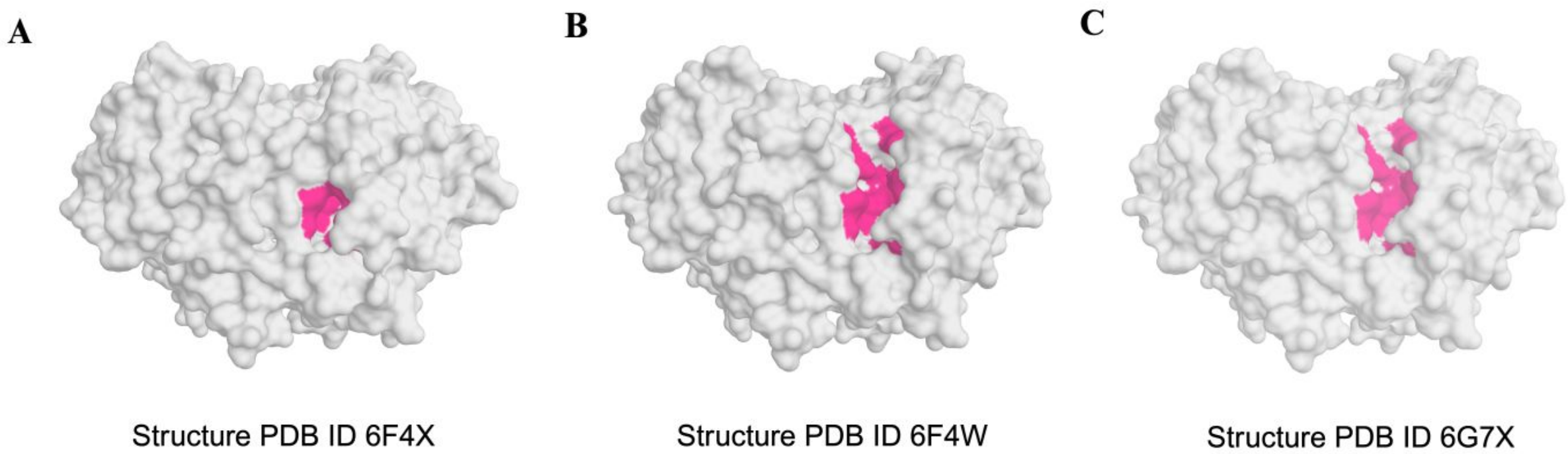

**Figure S3**. Docking poses of bromosphaerol (IPLSCC2) in the active site of *H. pylori* PNP across three receptor conformations, shown in molecular surface representation. (A) Overview of the hexameric enzyme with the binding region indicated; (B) closed conformation with a compact active site in the presence of a bound ligand (6F4X, chain C; wheat); (C) open conformation with bound ligand and the largest pocket volume (6F4W, chain D; light pink); (D) open apo conformation (6G7X, chain F; light blue). Surface representations illustrate the degree of compound burial within the active-site cavity in each conformational state, with greater burial observed in the open conformations (C, D) relative to the closed state (B).

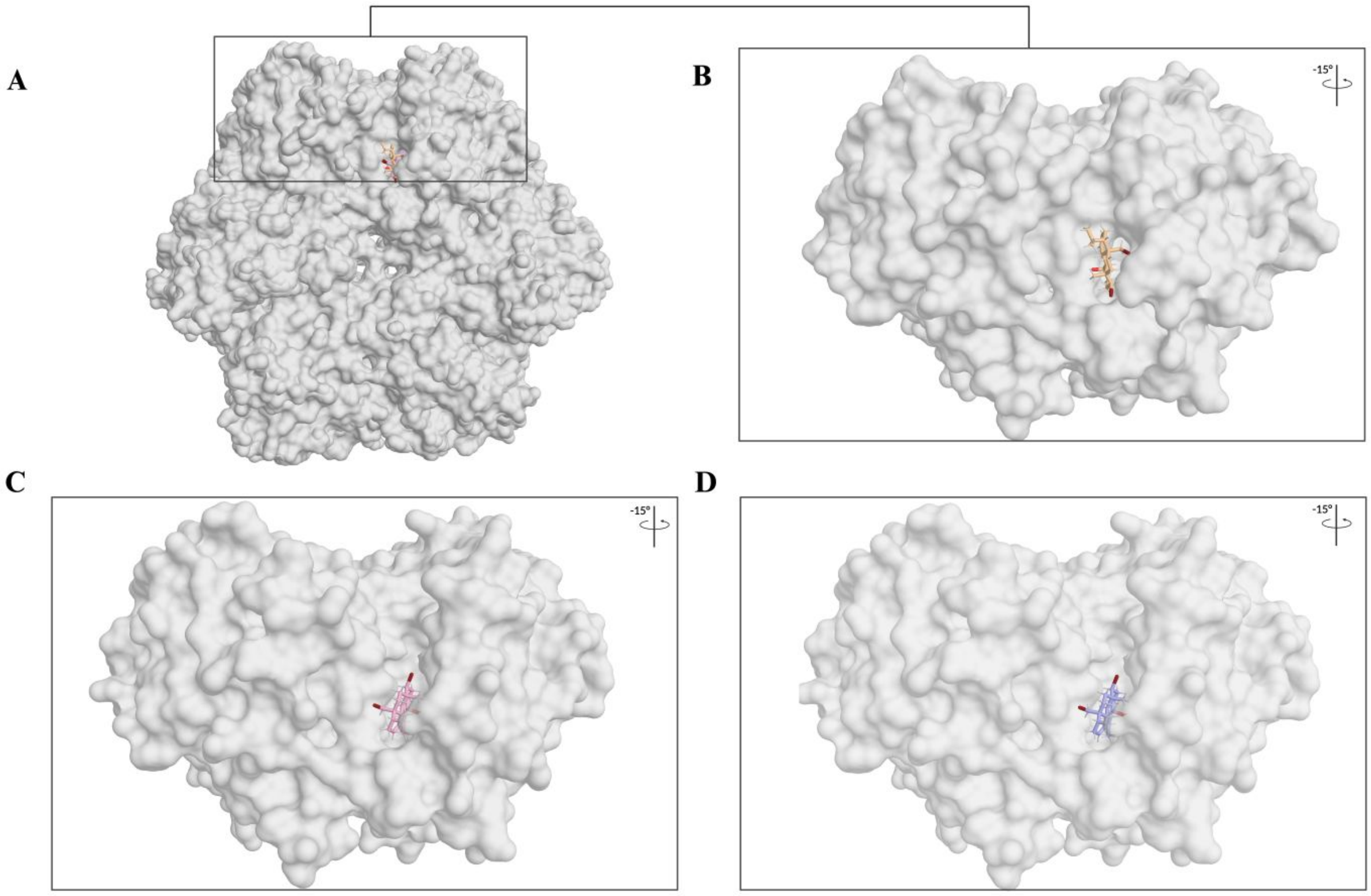

**Figure S4**. Docking poses of sphaerodactylomelol (IPLSCC6) in the active site of *H. pylori* PNP across three receptor conformations, shown in molecular surface representation. (A) Overview of the hexameric enzyme with the binding region indicated; (B) closed conformation with a compact active site in the presence of a bound ligand (6F4X, chain C; wheat); (C) open conformation with bound ligand and the largest pocket volume (6F4W, chain D; light pink); (D) open apo conformation (6G7X, chain F; light blue). Surface representations illustrate the degree of compound burial within the active-site cavity in each conformational state, with greater burial observed in the open conformations (C, D) relative to the closed state (B).

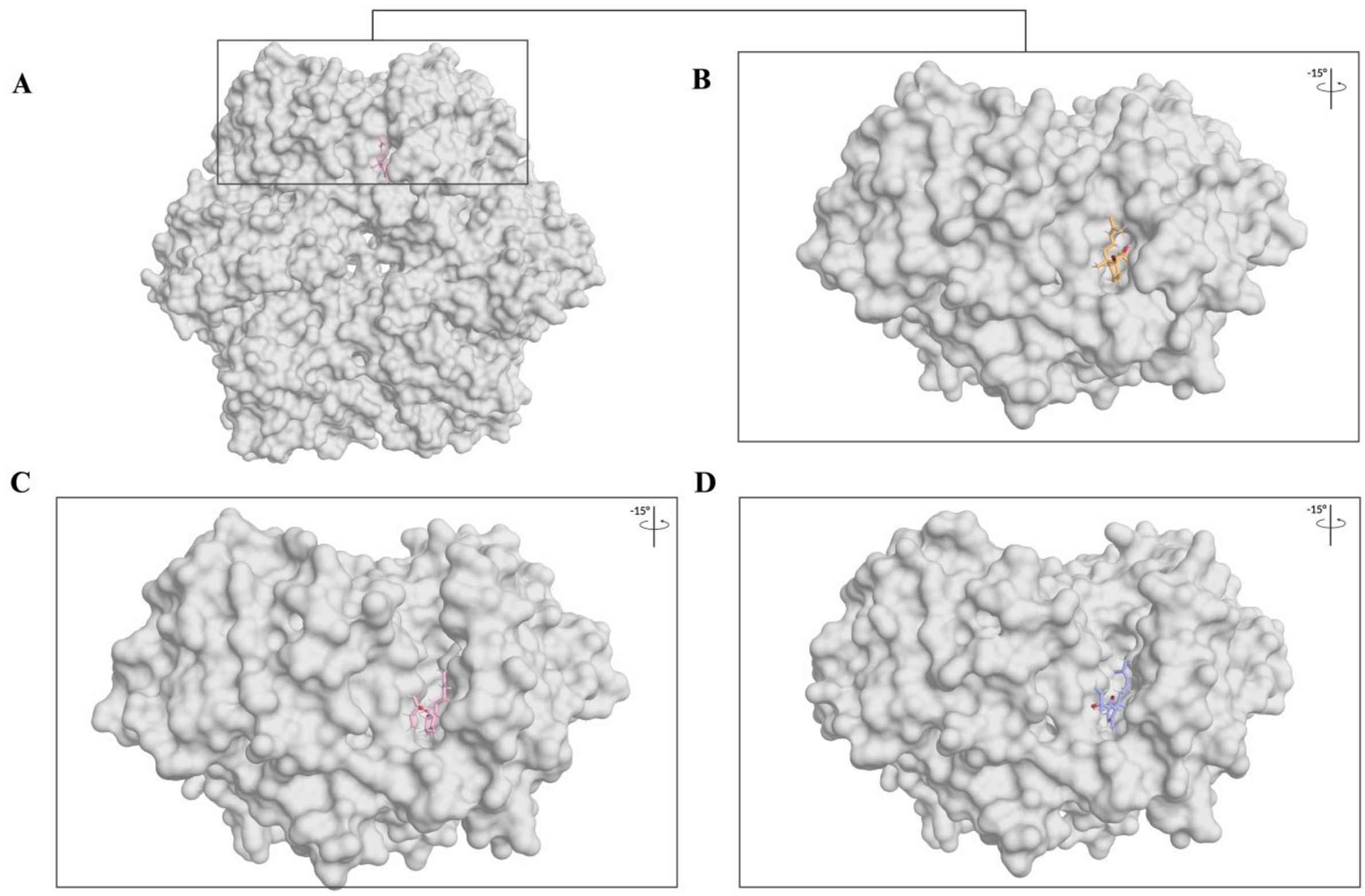

**Figure S5**. Docking poses of bromosphaerol (IPLSCC2) in the active site of *H. pylori* PNP using three receptor structures, shown in cartoon representation. **(A)** Overall view of the hexameric enzyme, with the region containing the active site highlighted; the boxed area corresponds to the region enlarged in the following panels. **(B)** Superposition of the docking poses obtained using the three selected receptor structures. **(C)** Superposition of the poses generated with the open structures 6F4W (chain D, light pink) and 6G7X (chain F, light blue), showing similar ligand positioning. **(D)** Docking pose generated with the closed structure 6F4X (chain C, wheat), showing a distinct orientation relative to the poses obtained with the open structures.

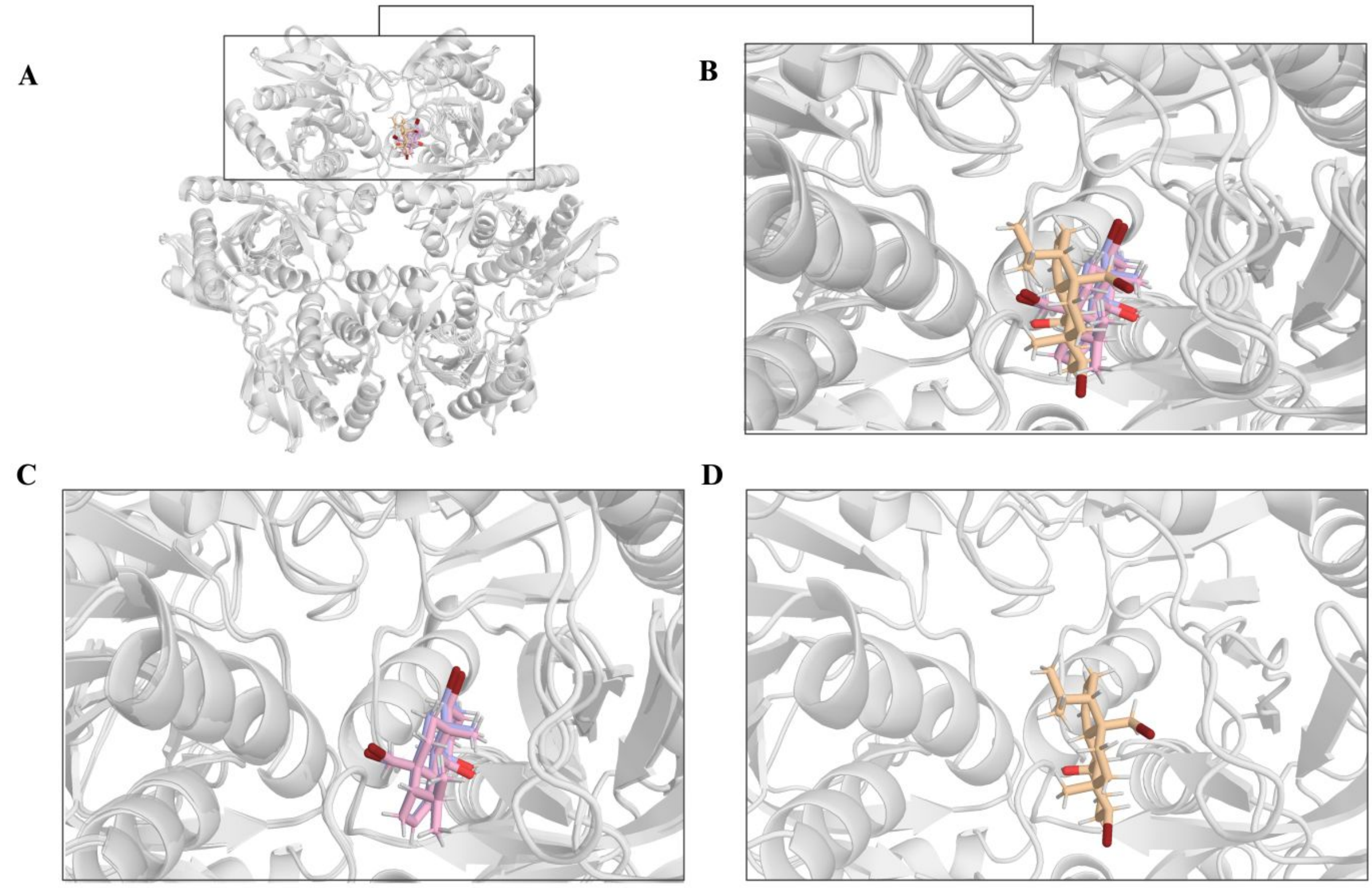

**Figure S6**. Docking poses of sphaerodactylomelol (IPLSCC6) in the active site of *H. pylori* PNP using three receptor structures, shown in cartoon representation. **(A)** Overall view of the hexameric enzyme, with the region containing the active site highlighted; the boxed area corresponds to the region enlarged in the following panels. **(B)** Superposition of the docking poses obtained using the three selected receptor structures. **(C)** Superposition of the poses generated with the open structures 6F4W (chain D, light pink) and 6G7X (chain F, light blue), showing similar ligand positioning. **(D)** Docking pose generated with the closed structure 6F4X (chain C, wheat), showing a distinct orientation relative to the poses obtained with the open structures.

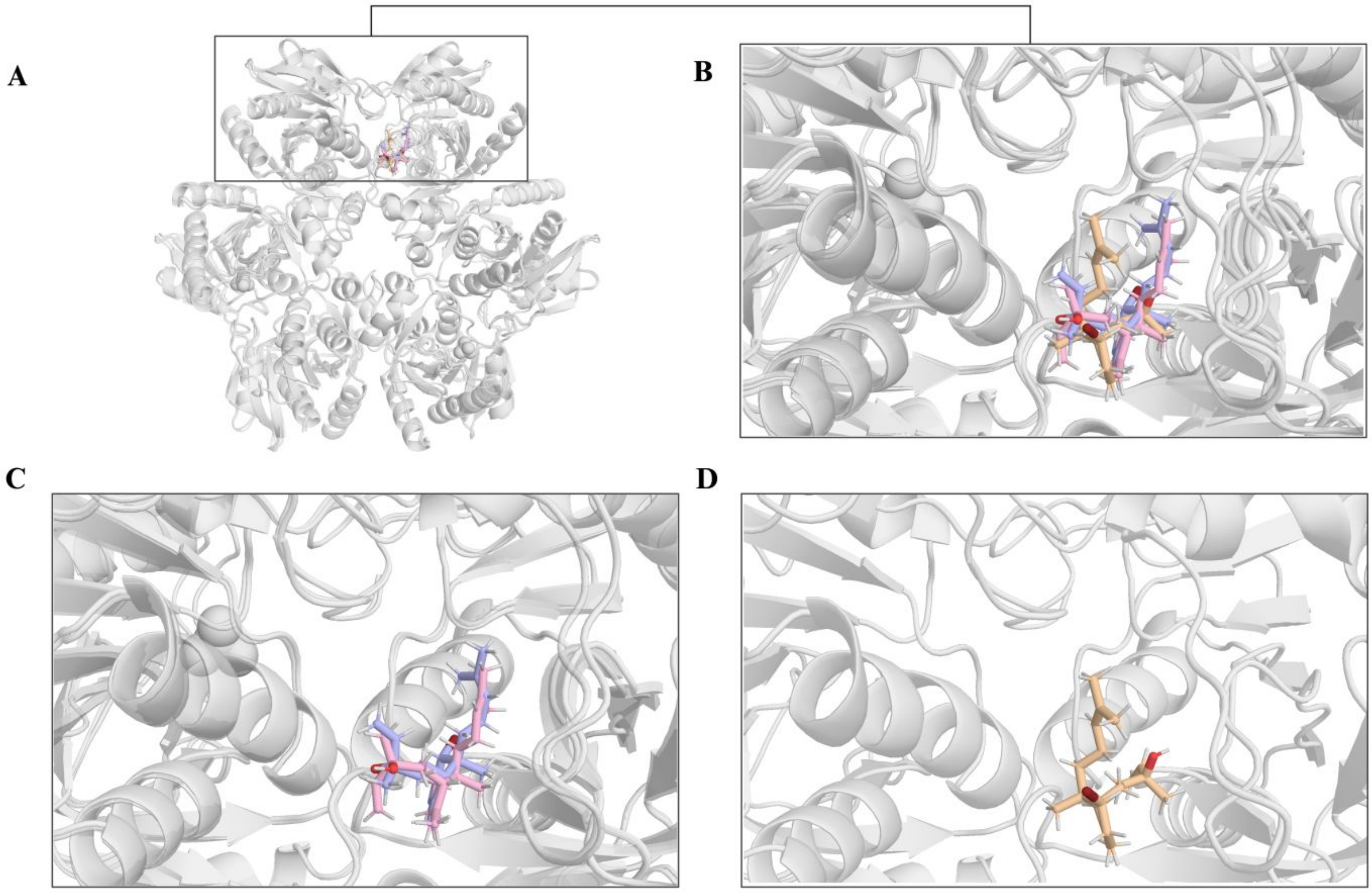

**Figure S7**. Docking poses of bromosphaerol (IPLSCC2) and sphaerodactylomelol (IPLSCC6) in the active site of *H. pylori* PNP, compared with the crystallographic formycin A, using the 6F4W structure, shown in cartoon representation. (A) Overall view of the hexameric enzyme, with the active site region highlighted by the box; this region corresponds to the area enlarged in panels B–D, which were rotated −50° around the Y-axis relative to the orientation shown in (A). (B) Formycin A (green), occupying a deeply buried position in the active site, as observed in the 6F4W crystallographic structure. (C) Superposition of the docking pose of bromosphaerol (light pink) with the crystallographic formycin A (green), showing partial overlap with the formycin A binding region, with the tricyclic scaffold extending toward a more peripheral portion of the pocket. (D) Superposition of the docking pose of sphaerodactylomelol (light blue) with the crystallographic formycin A (green), similarly showing partial overlap with the formycin A binding region, with its monocyclic core and flexible side chains extending toward a more peripheral portion of the pocket.

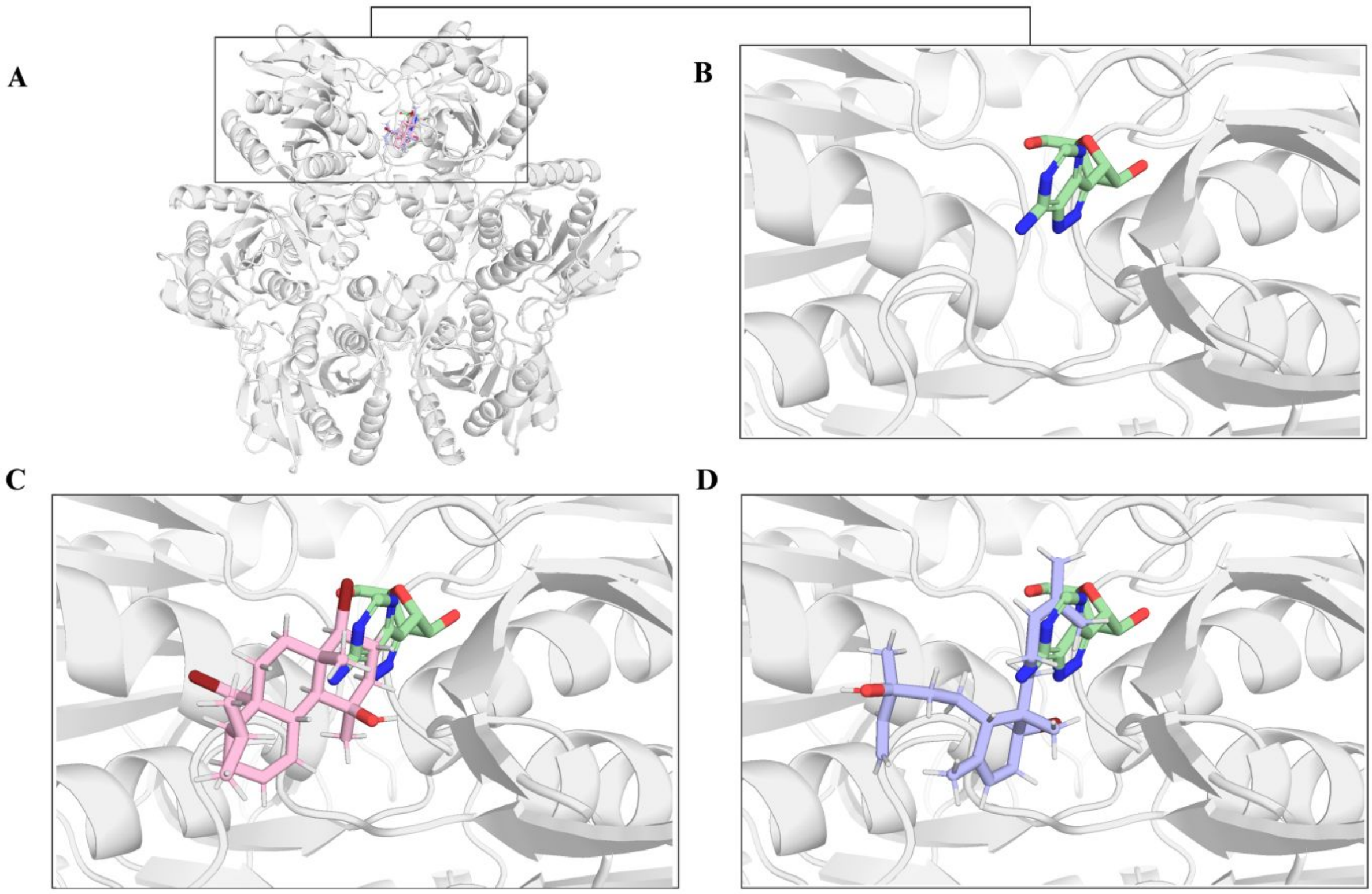

Supplement: Supplementary file 1 [file ao6c05168_si_001.pdf]
